# Analysis of the Usefulness of a Serious Game to Raise Awareness about Mental Health Problems in a Sample of High School and University Students: Relationship with Familiarity and Time Spent Playing Video Games

**DOI:** 10.3390/jcm8101504

**Published:** 2019-09-20

**Authors:** Adolfo J. Cangas, Noelia Navarro, José M. Aguilar-Parra, Rubén Trigueros, José Gallego, Roberto Zárate, Melanie Gregg

**Affiliations:** 1 Department of Psychology, Hum-760 Research Team, Health Research Centre, University of Almería, 04120 Almería, Spain; nng777@ual.es (N.N.); jgallego@ual.es (J.G.); 2 Department of Psychology, Hum-878 Research Team, Health Research Centre, University of Almería, 04120 Almería, Spain; 3 Department of Psychology, University of UCLA, Los Angeles, CA 90095, USA; rzarate@ucla.edu; 4 Physical Education Department, University of Winnipeg, MB 00000, Canada; m.gregg@uwinnipeg.ca

**Keywords:** serious games, stigma, mental health, education, inclusion

## Abstract

Background: One of the main challenges in the field of mental health today is the stigma towards individuals who have psychological disorders. Aims: This study aims to analyse the usefulness of applying a serious game developed for the purpose of raising awareness among students about mental health problems and analyse whether its usefulness can be influenced by the type of video games or the time that students usually devote to playing with this type of entertainment. Method: The serious game introduces four characters who display the symptoms of different psychological disorders. A total of 530 students participated in the study, 412 of whom comprised the experimental group and 118 the control group, 291 came from secondary school classes and 239 were university students. Results: The findings show that this serious game significantly reduced total stigma among students. Variables like time habitually spent playing video games or video game preference had no bearing on the results. Conclusion: Our findings suggest that the serious game is an appropriated tool to reduce stigma, both in high school and university students, independently of the type of video games that young people usually play, or time spent playing video games.

## 1. Introduction

One of the most important problems among young people today is the lack of knowledge about mental health issues. The World Health Organization [1] estimates that 10–20% of the world’s teenagers are at risk of developing various psychological disorders. For this reason, it is of utmost importance to promote good health to prevent and mitigate the effects of such illnesses.

Challenges to this important cause include the fact that many educators recognize that they are unable to detect or properly address these disorders among students [2]. Additionally, there is a great deal of doubt and confusion among students themselves regarding the truth about psychological disorders and what they can do to respond to them [3].

These issues are closely related to the social stigma that currently exists towards mental health disorders. This stigma is marked by widespread ignorance and erroneous information regarding psychological disorders. Moreover, ignorance and misinformation are not only limited to the general population but are also common among students, healthcare professionals, relatives of patients, and patients themselves [4,5,6]. This lack of understanding is a concern as stigma toward mental health can complicate treatment and rehabilitation [7].

In response to this reality, various public institutions are undertaking different programs to raise awareness about stigma in mental health. Most of these programs are based on three key strategies, which can be applied independently or in combination: (1) Provide information about psychological disorders, prognoses, and available resources for treatment; (2) facilitate contact with mental health patients so others have the opportunity to meet them personally, thereby dispelling many misconceptions; and, (3) organize public demonstrations to increase the visibility of the mental health population [8,9,10].

In terms of the format of intervention programs to help decrease stigma toward and increase awareness of psychological health, it is important to adapt to modern means of communication used by young people so they may more easily relate to the programs. For example, electronic resources, such as video games, are increasingly more popular among teenagers [11,12]. The use of serious games is also growing in a wide range of fields, including psychology, medicine, and education [13,14].

In the present case, the game, called Stigma Stop, depicts four characters who experience various psychological disorders (i.e., depression, agoraphobia, schizophrenia and bipolar disorder). Over the course of the game, players are provided with information about these illnesses and they are guided along as they learn about a variety of response strategies. Stigma Stop has been shown to be effective in reducing stigma among high school students. (i.e., secondary school and baccalaureate studies) between the ages of 14 and 18 [15]. In addition, with psychology students, it has been shown to be slightly superior in some dimensions to a talk by professionals and equivalent to a life history by mental health users [16]. However, secondary school and university students have not been compared jointly to determine whether significant differences exist between the two groups.

Similarly, one variable that may influence the usefulness or interest of students for the serious game is the time typically spent playing video games, or game preference, of the participants. For example, some studies have found that socially isolated individuals tend to prefer violent video games [17], but it is unclear whether spending more time playing video games or having a preference for a particular type of game can also determine the usefulness of a serious game among students, as in the case of Stigma-Stop, or if this same group would be interested in such a game.

In this way, the objectives of the present study were, firstly, to test the effectiveness of the serious game among secondary school, baccalaureate and university students and, secondly, to determine if the results can be influenced by familiarity and the type of video games that young people usually play.

## 2. Materials and Methods

### 2.1. Participants

The sample was selected using intentional non-probability sampling, as was a convenience sampling, using the classes themselves as individual units. The sample initially included 11 classes from five different high schools in the province of Almeria (Spain). These students participated during a series of scheduled school trips to visit and learn about the university. The remainder of the sample came from 11 randomly-selected classes from the School of Education (studying Early Childhood Education and Social Education) and the School of Social Sciences (studying Social Work and Psychology) at the University of Almería. The entire experiment was conducted in accordance with the Declaration of Helsinki. All participants also gave oral informed consent. Ethics approval was obtained from the Research Ethics Committee of the University of Almería, Spain. Students were only excluded from the sample if they refused to give their informed consent to participate. The participants received neither economic nor academic incentives for taking part in the study.

The final sample was comprised of a total of 530 participants (291 from secondary school and baccalaureate and 239 from the university). The age of the participants ranged between 14 and 59 years (M = 18.51; SD = 4.34). Regarding gender distribution, 326 were women and 204 were men. There were no significant gender distribution differences among the student groups (*p* > 0.05).

Of the entire sample, three classes from secondary school, two from the School of Social Sciences, and another from the School of Education were randomly assigned to the control group, while all others formed the experimental group. Thus, the control group was comprised of 118 students and the experimental group was comprised of 412 students. The experimental group was intentionally made larger than the control group, so more students could benefit from the intervention and because in previous studies we found that the responses of the control group were very stable [15,16]. Thus, the classes were randomly distributed: first, a class became a control group, then the next three were assigned to the experimental group, the next was designated as a control group and so on.

### 2.2. Instruments

#### 2.2.1. Questionnaire on Students Attitudes towards Schizophrenia (QSAS)

The QSAS [18] was initially developed in Germany and applied to secondary school students as part of the Global Program of the World Psychiatry Association against stigma and discrimination. The QSAS has 19 items that are rated on a three-point scale: agree, disagree and unsure. This instrument has been chosen because it is designed specifically for students and because schizophrenia is one of the most stigmatizing mental disorders [19]. The Spanish version of this questionnaire used in this study was validated by Navarro, Cangas, Aguilar-Parra, Moreno, Carrasco, Fuentes-Méndez and Gallego [20]; this version features two subscales (i.e., stereotypes and social distance and has been shown to have internal reliability with an overall Cronbach’s alpha of 0.95).

#### 2.2.2. Familiarity and Use of Video Games

It has been designed specifically for the present a questionnaire study asked participants to score the number of hours they typically devote to playing video games the last week (intervals of 0, 0–1 h, 1–3 h, 3–7 h, 7–14 h, 14–30 h or more than 30 h and the types of games they like most (i.e., choosing among the video game genres of action, platform, arcade, shooting, strategy, simulation, sports, racing, adventure, graphic adventures, virtual, musical or others). Students also have to indicate the usefulness and entertainment value of Stigma Stop (from 1 = not useful/entertaining to 10 = very useful/entertaining) and whether they would recommend it or not.

### 2.3. Intervention

Stigma-Stop [15] is a serious game that introduces four characters who experience various psychological disorders (i.e., depression, agoraphobia, schizophrenia and bipolar disorder). The player must interact with each character to gain their collaboration and, together, carry out a group task, which, in this case, is to participate in a video game design contest. During the different meetings, the characters begin to display characteristics that are common for their respective disorders. The game provides the player with information and offers various strategies on how to respond in the situations depicted. The player is initially asked to choose what they believe to be the most appropriate response among several possible options (feedback is offered for each of the alternatives presented). Additionally, the game interacts with participants by requesting them to give their opinion of the characters’ emotional states and by asking if they themselves have ever felt like the characters in the past or if they think they could help them. The objective is to foster empathy among participants towards individuals facing these challenges. Once interaction with each character has concluded, a mini-game is presented (i.e., memory, trivia, race, and shooting) whose content revolves around the topic of mental health.

### 2.4. Procedure

After giving informed consent the participants completed the QSAS [18] in a classroom while the researchers remained with the participants to administer the survey and to answer any questions. The researchers pointed out that all answers were anonymous and completely confidential. The students completed the questionnaire in a period of 10 min on average.

Next, Stigma Stop was applied to the group: four students in total from each class came forward to participate as volunteers who would try the serious game while the rest of the students followed the game’s progress on a projector screen in the classroom. 

Once the game had concluded, the participants were again given the QSAS [18] and the familiarity and use of video games questionnaire. 

The participants in the control group played other video games for one hour that were unrelated to the topic of mental health. They completed the QSAS, both before and after using the video games. 

## 3. Results

### 3.1. Data Analysis

To analyse the existence of statistically significant differences in the pre-test and post-test measurements of the two groups, the Student’s *t* was utilized for independent samples. This was complemented by an effect size with a statistic that corresponded, which in this case was Cohen’s *d*. In a second analysis, the post test and pre-test measurements of each group were compared using the Student’s *t* for related samples. The values considered in this study are those proposed by Cohen [21]): *d* = 0.20 (small), *d* = 0.50 (moderate) and *d* =0.80 (large), which is expanded by Rosenthal [22] with *d* = 1.30 (very large). In relation to the different groups of students we determine, through the Student’s *t* value, if statistically significant differences exist between high school and university students in the pre-test and post-test for all the variables, as well as the total level of stigma, including the effect size with Cohen’s *d*. These observations led to an intra-group analysis to determine whether the program had an effect between the pre-test and post-test in each of the subgroups. Finally, a multivariate analysis of variance (MANOVA) was carried out to evaluate the influence that education level and gender had on the benefits of the intervention. In addition, the analyses were completed with Pearson’s correlations, on one hand, between the time spent playing video games and scores for Stigma Stop’s entertainment and usefulness and, on the other hand, between types of games, the number of hours spent playing and scores for the game’s usefulness and entertainment. The analysis was carried out using the statistics program SPSS 24.0 (IBM, Armonk, NY, USA).

### 3.2. Reduction of Stigma

As can be seen in Table 1, the pre-test measurements for the experimental group and the control group do not reveal the existence of statistically significant differences for the analysed variables: social distance and stereotypes. However, statistically significant differences between the two groups were found for these two variables and the total score after the intervention. Cohen’s *d* allowed us to confirm that the size of the effect was moderate in both the social distance subscale and the total score, and low in the stereotypes subscale. 

Table 2 displays the mean scores and standard deviations of the subscales for the experimental and control groups at pre and post intervention. The analysis of pre-test and post-test scores for the control group revealed no statistically significant differences in the dependent variables. However, significant differences were observed for the same analysis with the experimental group, both in terms of the total questionnaire score and the two subscales: social distance and stereotypes. Therefore, the levels of stigma decreased among the participants who were exposed to Stigma Stop; this stigma reduction was more marked on the social distance subscale compared to the stereotypes subscale. 

In relation to the different groups of students, it can be seen in Table 3 that statistically significant differences exist between high school and university students in the pre-test for all the variables as well as the total level of stigma, including a considerable effect size. These differences between the two groups are also present in the post-test, although they are not as large, as indicated by the effect size. These observations led to an intra-group analysis to determine whether the program had an effect between the pre-test and post-test in each of the subgroups (Table 4). It is observed that university students lowered stigma in relation to all the variables, yet only the reductions in social distance and the total score were significant while the effect size for stereotypes was moderate. However, in the case of the secondary school students, stigma was significantly reduced in all the variables, with a strong effect size in terms of social distance, moderate for the total score, and low for stereotypes. Thus, stigma was reduced to a greater extent among the secondary school students following the intervention, demonstrating the program had a greater effect on this population. 

Finally, a multivariate analysis was carried out to evaluate the influence that education level and gender had on the benefits of the intervention. The multivariate analysis of variance (MANOVA) determined that there were statistically significant differences due to education level (*p* < 0.001, *F* (2.000) = 19.031, Lambda de Wilks = 0.914; *η^2^* = 0.086), albeit in this case the size of the effect was small according to this eta squared. In contrast, the differences based on gender were not significant (*p* = 0.626, F (4.000) = 0.652, Wilks’s lambda = 0.994; *η^2^* = 0.003), and neither was the interaction between gender and education level (*p* = 0.495, F (2.000) = 0.704, Lambda de Wilks = 0.997; *η^2^* = 0.003). Having obtained these results, it could be concluded that the effect of the program was the same for all participants independent of gender but not in terms of education level (university or secondary school and baccalaureate), albeit, in the case of education, the effect size indicated that the differences were small.

### 3.3. Relationship with Familiarity and Time Spent Playing Video Games

With regard to the participants’ evaluation of the game itself, the students gave a high score for usefulness (8.13 out of 10 possible points) and a slightly lower score for entertainment value (6.86). Additionally, 91% of participants said they would recommend playing Stigma Stop to a friend. This assessment was higher among high school students (8.5 for usefulness and 7.4 for entertainment value; 99% would recommend it to a friend) than among university students (7.9 for usefulness and 6.6 for interest; 88% would recommend it to a friend). 

Regarding the amount of time that students spend playing video games, it is observed that time bears no relationship with the usefulness and entertainment value of the program Stigma Stop (Table 5). 

Similarly, regarding the types of games young people like best, it is observed that some types (e.g., action, platform, shooting, and sports) positively correlate with time spent playing games. However, none of them were found to have a statistically significant relationship with the usefulness or entertainment value of Stigma Stop (Table 6).

## 4. Discussion

After applying Stigma Stop to a sample consisting of both high school and university students, it was confirmed, as in previous studies [15,16], that the serious game helped to reduce stigma in the total sample, this reduction did not occur in the control group. The stigma factor that was most sensitive to these changes was the variable social distance, while the decrease was less for the variable stereotypes. These findings are important when considering that the image of violence and threat is the most common stigmatizing notion associated with severe psychological disorders [23]. 

When the sample is divided between high school students and university students, a slightly higher effect is observed for the former group. It is also shown that high school students possessed higher initial stigma values. While stigma was significantly lowered in the university students, the effect was slightly smaller compared to the high school students as the university students initially displayed lower levels of stigma.

With regard to the university students, it is observed that stigma was lower in the pre-test evaluation, revealing that more years of education may have an influence on the reduction of stigma [24]. Nonetheless, as various studies confirm, university students also possess stigmatizing ideas similar to those of the general population, although to a lesser extent, and this is even the case among students enrolled in healthcare studies, such as medicine and nursing [25,26]. Thus, there is evidently a need to carry out interventions that specifically target this group [25,27]. Furthermore, many professionals working in the education sector recognize that they lack the training to properly respond to the needs of individuals with psychological disorders [28], revealing that they also require tools that would be useful in their field.

With regard to the demographic variables, it was observed that Stigma Stop had a similar effect among male and female students. No significant differences were found in the results following the intervention, neither when analysed separately nor when gender was evaluated in combination with education level. 

As for the students’ evaluation of the game, they gave it a high score for its usefulness and a slightly lower score for its entertainment value. Most participants also stated they would recommend the game to a friend. In this regard, slightly higher scores were registered among high school students than university students. 

Regarding the effect that familiarity and time students habitually spend playing video games can have, we found no significant relationship. This finding may own to the fact that Stigma-Stop is a serious game, that is, a game with educational purposes, and students understand that it is “different” from games whose only purpose is to entertain. Indeed, students gave the game a higher score for usefulness than for entertainment. In any case, in future studies it would be interesting to examine this point further, perhaps resorting to a qualitative methodology that could provide more information on the opinion of the participants related to these aspects [29]. 

Pertaining to the limitations of this study, we acknowledge that no follow-up on the results was conducted to see if the reduction in stigma persisted over time. Moreover, the sample of university students was comprised of only individuals from the Schools of Education and Social Sciences and it would be prudent to carry out the intervention with other degree programs as well. Similarly, the number of participants in the control group was lower than in the experimental group. 

Finally, as for future research lines, we believe that it is very important that new works focus on stigma towards disorders other than schizophrenia, such as the other illnesses presented in Stigma-Stop (i.e., depression, agoraphobia and bipolar disorder). Furthermore, it would be beneficial to analyse the combined effect of applying a serious game in conjunction with other traditional interventions (such as a talk by a professional or direct contact with mental health patients). 

## Figures and Tables

**Table 1:** Pre-test and post-test differences between the experimental group and the control group.

		Pre Test			Post Test	
	*t*	*p*	*d*	*t*	*p*	*d*
Social distance	0.94	0.34	0.070	−4.96	0.00	−0.50
Stereotypes	−1.57	0.11	−0.15	−2.45	0.015	−0.26
Total stigma	−0.11	0.91	−0.01	−4.24	0.00	−0.41

Note: *t* = *t*-students; *p* = *p*-value; *d* = *d*-Cohen

**Table 2:** Mean scores, standard deviations, and *t* test at pre- and post-intervention.

	Experimental					Control				
	Pre (*n* = 412)	Post (*n* = 412)	Pre-Post			Pre (*n* = 118)	Post (*n* = 118)	Pre-Post		
	M (SD)	M (SD)	*t*	*p*	*d*	M (SD)	M (SD)	*t*	*p*	*d*
Social distance	4.78 (2.56)	3.10 (2.48)	13.70	0.00	0.66	4.26 (9.30)	4.33 (2.35)	−0.09	0.93	−0.01
Stereotypes	4.02 (3.39)	3.53 (3.13)	3.77	0.00	0.15	4.58 (3.78)	4.46 (3.88)	−0.74	0.45	0.03
Total stigma	8.77 (5.22)	6.64 (4.83)	10.78	0.00	0.42	8.85 (10.72)	8.80 (5.55)	−0.05	0.95	0.01

Note: M = Mean; SD = Standard Deviation; *t* = *t*-students; *p* = *p*-value; *d* = *d*-Cohen.

**Table 3:** Mean differences between the pre-test and post-test of high school and university students.

		Pre Test			Post Test	
	*t*	*p*	*d*	*t*	*p*	*d*
Social distance	−8.73	0.00	−0.86	−6.96	0.00	−0.67
Stereotypes	−7.22	0.00	−0.70	−5.20	0.00	−0.50
Total	−9.21	0.00	−0.90	−7.08	0.00	−0.68

Note: *t* = *t*-students; *p* = *p*-value; *d* = *d*-Cohen.

**Table 4:** Pre-test/post-test differences among high school students and university students.

	University Students					High School Students				
	Pre Test (*n* = 181)	Post Test (*n* = 181)	Pre-Post			Pre Test (*n* = 231)	Post-Test (*n* = 231)	Pre-Post		
	M (SD)	M (SD)	*t*	*p*	*d*	M (SD)	M (SD)	*t*	*p*	*d*
Social distance	3.59 (2.29)	2.22 (1.90)	9.11	0.00	0.65	5.62 (2.39)	3.78 (2.65)	10.31	0.00	0.72
Stereotypes	2.76 (2.65)	2.68 (2.22)	0.46	0.64	.0032	4.98 (3.58)	4.17 (3.53)	4.38	0.00	0.22
Total	6.35 (4.24)	4.90 (3.42)	5.84	0.00	0.37	10.61 (5.14)	7.96 (5.29)	9.12	0.00	0.50

Note: M = Mean; SD = Standard Deviation; *t* = *t*-students; *p* = *p*-value; *d* = *d*-Cohen.

**Table 5:** Correlations between the time spent playing video games and ratings of Stigma Stop’s entertainment and usefulness.

		Entertaining	Useful	Time
Entertaining	Pearson Correlation			
	Sig. (bilateral)			
Useful	Pearson Correlation	0.67 **		
	Sig. (bilateral)	0.00		
Recommend	Pearson Correlation	−0.40 **	−0.36 **	−0.062
	Sig. (bilateral)	0.00	0.00	0.42
Time	Pearson Correlation	0.10	0.00	
	Sig. (bilateral)	0.17	0.90	

Sig., Significant correlation; **, Significant correlation at 0.01 level (bilateral).

**Table 6:** Correlations between types of games, number of hours spent playing and Stigma Stop ratings for usefulness and entertainment.

		Entertaining	Useful	Time
None	Pearson Correlation	0.02	0.11	0.26 **
	Sig. (bilateral)	0.78	0.15	0.00
Action	Pearson Correlation	−0.12	−0.09	−0.23 **
	Sig. (bilateral)	0.12	0.24	0.01
Platform	Pearson Correlation	0.01	0.01	−0.23 **
	Sig. (bilateral)	0.95	0.94	0.01
Arcade	Pearson Correlation	0.09	0.08	−0.11
	Sig. (bilateral)	0.23	0.28	0.13
Shooting	Pearson Correlation	0.03	−0.05	−0.26 **
	Sig. (bilateral)	0.67	0.51	0.00
Strategy	Pearson Correlation	0.02	0.08	−0.11
	Sig. (bilateral)	0.79	0.31	0.12
Simulation	Pearson Correlation	−0.09	−0.09	−0.06
	Sig. (bilateral)	0.23	0.23	0.40
Sports	Pearson Correlation	0.00	−0.10	−0.13
	Sig. (bilateral)	0.99	0.18	0.078
Racing	Pearson Correlation	−0.05	−0.15	0.040
	Sig. (bilateral)	0.51	0.05	0.59
Adventure	Pearson Correlation	−0.13	−0.10	−0.09
	Sig. (bilateral)	0.07	0.19	0.23
Adventure Graphics	Pearson Correlation	0.01	−0.04	0.03
	Sig. (bilateral)	0.84	0.57	0.65
Virtual Worlds	Pearson Correlation	−0.10	0.02	−0.05
	Sig. (bilateral)	0.18	0.76	0.50
Musical	Pearson Correlation	−0.02	−0.04	−0.04
	Sig. (bilateral)	0.78	0.61	0.57
Others	Pearson Correlation	0.02	−0.02	−0.08
	Sig. (bilateral)	0.82	0.83	0.32

Sig., Significant correlation; **, Significant correlation at 0.01 level (bilateral).
